# Blends of Carboxymethyl Cellulose and Cottonseed Protein as Biodegradable Films

**DOI:** 10.3390/polym16111554

**Published:** 2024-05-31

**Authors:** Huai N. Cheng, Atanu Biswas, Gary Kuzniar, Sanghoon Kim, Zengshe Liu, Zhongqi He

**Affiliations:** 1 USDA Agricultural Research Service, Southern Regional Research Center, New Orleans, LA 70124, USA; 2 USDA Agricultural Research Service, National Center for Agricultural Utilization Research, Peoria, IL 61604, USA

**Keywords:** CMC, cottonseed meal, cottonseed protein, polyblend, water-soluble films, food packaging, dissolvable bags

## Abstract

With the increasing awareness of plastic pollution in the environment and the accumulation of microplastics in water, a significant amount of research and development is ongoing to replace the synthetic plastics in packaging and coatings. In this work, we explored the blends of carboxymethyl cellulose (CMC) and washed cottonseed meal (CSM, consisting mostly of cottonseed protein) as agro-based, biodegradable, and sustainable alternatives to plastics. Glycerol was found to be a suitable plasticizer for these blends. The blends of CMC/CSM were produced as single-layer films from 50 to 90 μm in thickness, consisting of different proportions of the components and plasticizer. The evaluated properties included opacity, water vapor permeability, mechanical properties, thermogravimetric analysis, moisture sorption analysis, and water swelling test. Higher percentages of CSM in the blend resulted in higher opacity and lower water vapor permeation rates. The mechanical strength waned with lower levels of CMC. Possible applications for these blends include their use as water-soluble food packaging and coatings and as dissolvable bags and pouches for detergents and agrochemicals.

## 1. Introduction

Conventional plastic packaging, primarily derived from non-renewable fossil fuels, poses significant environmental challenges [1,2]. The durability of plastics, combined with their slow degradation rate, leads to their deposition in landfills and natural environments. Moreover, microplastics can accumulate in water. This type of pollution not only disrupts ecosystems but also harms wildlife through ingestion and entanglement. Moreover, the production of conventional plastics contributes to greenhouse gas emissions and depletes valuable resources.

Biodegradable polymers are typically derived from renewable sources such as plants, bacteria, or algae. The use of biodegradable polymers for food packaging offers several advantages [3,4]. First, biodegradable polymers reduce environmental pollution. Unlike conventional plastics, they break down naturally over time, minimizing the accumulation of waste in landfills and oceans. This characteristic alleviates the burden on the ecosystem and reduces the risk of harm to wildlife. Secondly, biodegradable polymers promote sustainability. By utilizing renewable sources as feedstock, such as plant-based materials, the reliance on non-renewable fossil fuels diminishes. This shift supports sustainable agriculture and reduces the carbon footprint associated with packaging production. Thirdly, if the biodegradable polymers are water-soluble, they can serve as soluble films and coatings in food and related applications.

While the use of biodegradable polymers in food packaging is promising, certain challenges and considerations need to be addressed [5,6,7]. A key consideration is the cost of production for biodegradable polymers, which is relatively higher than conventional plastics due to limited economies of scale. However, as technological advancements and market demand increase, prices are expected to become more competitive. Additionally, the biodegradable polymers need to exhibit satisfactory food packaging properties, and the production of biodegradable polymers does not compete with food production or contribute to deforestation.

Carboxymethyl cellulose (CMC) is a commercially available, water-soluble cellulosic derivative. It is commonly used as a viscosity modifier, thickener, emulsion stabilizer, film-former, suspension aid, and drilling fluid [8,9]. It has previously been studied for food packaging applications [10,11,12] and is known to be biodegradable [10]. Cottonseed protein is derived from the kernel of cottonseed [13] and has been considered for use as a bioplastic [14,15,16,17], wood adhesive [18,19], and packaging film in combination with a plasticizer [20,21]. As a naturally occurring protein, it is biodegradable [22]. In previous work [23,24], cottonseed meal (CSM), suitably washed with water or buffer, was found to be inexpensive and could contain up to 70% protein; it was as effective as cottonseed protein isolate in wood adhesive studies.

There is currently a substantial market (estimated to be of USD 364 million in 2020) for water-soluble films, primarily for poly(vinyl alcohol) [25]. It may be useful to consider an agro-based alternative. In this work, we have made water-soluble films from the blends of CMC and CSM and measured their properties. The role of glycerol as a plasticizer has also been demonstrated. In addition, moisture sorption analysis and water swelling tests were also carried out. The purpose of this is to assess the possibility of these films as water-soluble packaging materials. As far as the authors are aware, there have not been previous efforts to use blends of CMC and cottonseed protein as soluble films or coatings. 

## 2. Materials and Methods

### 2.1. Materials

CMC (degree of substitution of 0.7), glycerol, sodium hydroxide, and poly(vinyl alcohol) (99% hydrolyzed) were purchased from Sigma-Aldrich, Milwaukee, WI, USA. CSM was provided by Cotton Inc. (Cary, NC, USA). The washing procedure [23,24] entailed grinding the CSM to <0.5 mm in particle size, mixing with water, centrifuging (6000 rpm, 15 min) to retrieve the solid, repeating the water mixing and centrifuging, and then drying and milling. The final washed CSM had about 65% protein content, with mostly carbohydrates and lesser amounts of lignin and inorganics that made up the composition. 

### 2.2. Preparation of Films

For film preparation, a modified method from the work of Biswas et al. [11] was employed. First, CSM was dissolved at about 3% concentration in distilled water, with the pH adjusted to 10.5 using NaOH. The solution was filtered to remove insoluble materials. Separately, CMC was dissolved in distilled water at 2%. Both stock solutions were heated at 80 °C to fully dissolve, if needed. For each formulation, the two stock solutions were added together in the proper ratio so that the correct dry weight ratio was obtained, as given in Table 1. The total solution should be approximately 1% (*w*/*v*) concentration. If it was not, more distilled water was added so that all samples were at 1% concentration. The pH of the final solutions was adjusted to 10.5 using NaOH. Then, 0, 20, or 40% glycerol (based on total CMC/CSM weight) was added as plasticizer and stirred at 70 °C. Any bubbles in the filmogenic solutions were removed using a vacuum pump. 

The filmogenic solutions were cast into films on 10″ × 15″ polypropylene trays; any small bubbles were dispersed to give clear solutions. The baking dishes were then put in a forced air oven at 35 °C until dry. At least five replicates of each of the formulations were made. After the films were removed from the dishes, they were conditioned in a temperature- and humidity-controlled room (23 ± 1 °C and 50 ± 5% RH) for at least 40 h before mechanical testing. 

### 2.3. Fourier Transform Infrared (FT-IR) 

The instrument used for FT-IR was a Perkin-Elmer Frontier spectrometer (Waltham, MA, USA). The instrumental conditions included ATR mode at room temperature, spectral region 650–4000 cm^−1^, 32 scans per spectrum, and 4 cm^−1^ resolution. The collected data were transferred from the instrument to a computer and processed with an Excel spreadsheet (Version 2404, Microsoft, Redmond, WA, USA).

### 2.4. Mechanical Properties of the Films

The thicknesses of test specimens were measured at five different locations of the film with an ElectroPhysik minitest (Model No. 3100, Dr. Steingroever GmbH & Co., KG, Cologne, Germany), and the average values were used. Young’s modulus (YM), tensile strength (TS), and elongation at break (EB) were measured for each sample using an Instron Universal testing system (Model 3365, Instron Corp., Norwood, MA, USA; Bluehill Universal software, Version 4.25). The initial gauge length was set at 250 mm, and the rate of grip separation was 25 mm/min [26], using a 1 kN load cell. Each test was repeated with five film strips, and the result was reported as average ± standard deviation. 

### 2.5. Water Vapor Permeability (WVP) 

The WVP was determined gravimetrically, based on the ASTM E96-00 method [27], using an ESPEC humidity chamber (Model EPL-3H, Hudsonville, MI, USA). The films were cut (discs with a diameter of 50 mm) and placed at the top of a permeation cell (Elcometer, Warren, MI, USA, model 5100) containing a desiccant. The cell was then placed in a humidity chamber at 23 °C and 50% RH. The cell was weighed over a 48 h period and data were recorded at intervals of at least 1 h. The calculations were carried out according to the ASTM method [27].

### 2.6. Opacity

The opacity measurement was based on the ASTM method D1746-92 [28] and determined with a Shimadzu UV–Vis spectrophotometer (model 2600, Norwood, MA, USA), with UV Probe 2.43 software. A film rectangle measuring 400 mm long and 100 mm wide was adhered to the cuvette mount. Each sample was measured between 400 and 800 nm for the absorption range of visible light. Calibration for 100% transmittance was performed using a cuvette mount without a sample. Film opacity was defined as the absorbance at 550 nm divided by the film thickness. Opacity was then expressed as absorbance units/mm (A-mm^−1^). Three measurements were made for each sample and averaged.

### 2.7. Thermal Analysis

Thermogravimetric analysis (TGA) was performed using a Q500 TGA instrument (TA Instruments, New Castle, DE, USA). Each sample (~5 mg) was weighed into a tared, open platinum TGA pan and measured in a nitrogen atmosphere by heating at 10 °C/min up to 600 °C. The data were analyzed for weight loss and derivative thermogravimetry (DTG) modes using the Universal Analysis software program (Version 4.5A) from TA Instruments.

### 2.8. Optical Microscopy

Optical microscopic images of the films were obtained with an inverted microscope (Model Axiovert 100, Zeiss, Waltham, MA, USA) equipped with a phase-contrast objective lens (×10). A small piece of each film (5 mm × 10 mm) was placed in between two slide glasses, which were tightly bound together with adhesive tape. The phase contrast images were taken by a digital camera (Model IMX183; AmScope, Irvine, CA, USA). The images obtained were transferred to a computer and processed with image software (Preview v. 11.0; Apple Inc., Cupertino, CA, USA) for optimum brightness/contrast control.

### 2.9. Moisture Sorption Analysis

The adsorption of water on the films was measured using a Q5000 SA Dynamic Vapor Sorption analyzer (TA Instruments, New Castle, DE, USA). A small square (5 mm × 5 mm) of each sample film was cut and placed in a tared sample pan. Each sample was equilibrated at 25 °C and 0% relative humidity (RH) for 6 h. The humidity level was then increased at 10% increments from 0 to 90% RH, then back to 0% RH in 10% increments, holding at each step for 6 h. This allowed both water adsorption and desorption of each film to be observed. Data analysis and sorption isotherm results were obtained using the Universal Analysis 2000 software (version 4.5A, TA Instruments, New Castle, DE, USA).

### 2.10. Swelling Test

A water swelling test was performed through a procedure that was adapted from part 7.4 of the ASTM D570-98 water swelling test for polymer films [29]. Each dry film was weighed and placed on a filter paper in a Buchner funnel with attached vacuum suction. Water was added to the Buchner for a specified time interval (from 0 to 60 s) to allow for the soaking of the film and removed through vacuum suction at the end of the time interval. The film was gently turned over on the filter paper to remove excess water and weighed. The weight ratio was obtained from the weight of the swollen film and the weight of the dry film. 

## 3. Results and Discussion

A total of 11 films containing CMC, CSM and glycerol were made and evaluated as possible packaging films, as shown in Table 1. Evaluation was carried out via mechanical testing, water vapor permeability, opacity, FT-IR, TGA, optical microscopy, moisture sorption analysis, and water swelling test.

### 3.1. Mechanical Properties

The mechanical properties of the films are shown in Table 2. The CMC films without CSM and glycerol (sample A1) show the largest YM (3909 MPa) and TS (56 MPa) but relatively low EB (5%). For CMC/CSM blends (comparing samples A1, A2 and A3), YM, TS, and EB all decrease with CSM addition. When glycerol is added to CMC (comparing samples A1, B1, and C1), YM and TS decrease notably, but EB increases. Thus, glycerol acts as a plasticizer for CMC. 

For CMC:CSM blends (75:25 or 50:50), the addition of glycerol shows the same trends: YM and TS are reduced, but EB is increased. It is of interest that the combination ratio of 75:25:40 for CMC:CSM:glycerol (sample C2) gives the maximum value for EB, but a moderate value for TS. Thus, for an application having specifically allowable values of YM, TS, and EB, a suitable combination of CMC:CSM: glycerol blend may be selected for film-making. 

In the literature, mechanical data for blends of CMC/gelatin and CMC/soy protein were previously published. For CMC/gelatin blends [30], CMC showed a larger TS and a larger YM than gelatin, and the blends were intermediate in values, similar to our data for CMC/CSM blends. Likewise, the CMC/soy protein blends gave higher TS values than soy protein alone [31,32,33]. However, the EB values were more variable. In one study [32], the CMC/soy protein blends showed greater EB values than soy protein alone, whereas in another study [33], the opposite trend was found. For CMC/gelatin blends, higher gelatin content gave higher EBs [30] because gelatin film was more flexible.

### 3.2. Water Vapor Permeation (WVP) and Opacity

The WVP data for the CMC/CSM blend films are shown in Table 3. CMC (sample A1) shows the highest WVP value. Blending with CSM (samples A2 and A3) reduces the WVP. The addition of 20 parts by weight of glycerol (samples B1, B2, and B3) also reduces the WVP of CMC and CMC/CSM blends. However, at 40 parts by weight of glycerol, the WVP values tend to be similar. Thus, the blending with CSM provides CMC with a modest improvement in moisture barrier properties. A similar trend was shown for CMC/soy protein blends [34], where a higher CMC content gave a higher WVP value. 

The corresponding opacity data are shown in the last column in Table 3. CMC is relatively transparent with an opacity of 1.2 A/mm. The addition of CSM to CMC (samples A2 and A3) noticeably increases the opacity. The addition of glycerol slightly reduces the opacity of all the samples. This observation is consistent with CMC/soy protein blends, where a higher soy protein content was found to give a higher opacity [32]. In general, high opacity is advantageous in food packaging as it can potentially minimize light-induced reactions in food products. However, in certain scenarios, a lower opacity film may be preferred when consumers wish to inspect the food item inside the package before buying. Hence, films with varying levels of opacity offer a broader selection for specific applications as required.

### 3.3. FT-IR Data

**Figure 1:**
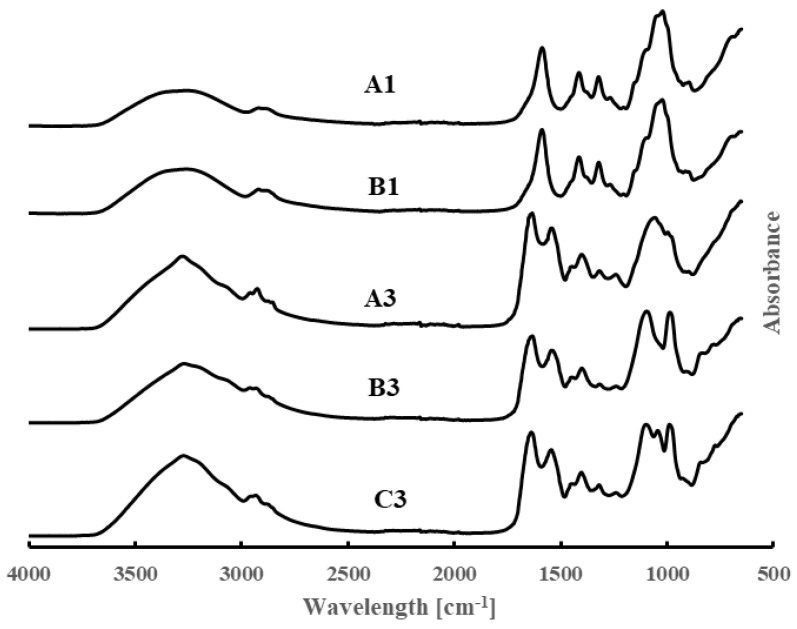
FT-IR spectra of samples A1, B1, A3, B3, and C3 (from top to bottom).

The FT-IR data for samples A1, B1, A3, B3, and C3 are shown in Figure 1. For sample A1 (pure CMC), the peaks at 3200–3600 cm^−1^ correspond to O–H stretching, 2840–3000 cm^−1^ due to C–H stretching, 1650–1780 cm^−1^ due to carboxyl stretching, 1600–1650 cm^−1^ and 1420 cm^−1^ due to carboxylate anion stretching, 1640 cm^−1^ due to water, 1329 cm^−1^ due to -OH bending, and ca. 1000 cm^−1^ due to various C–O, C–O–H, and C–O–C modes [35,36,37]. In sample B1 (containing CMC and glycerol), the glycerol IR peaks occur at 3260 cm^−1^ (broad band) for O–H stretching, 2931 and 2879 cm^−1^ for C–H stretching, 1200–1460 cm^−1^ for various C–H, O–H, and CH_2_ modes, and 990–1150 cm^−1^ for C–O and C–O–H vibrations [38,39,40]. These peaks may be shifted somewhat due to hydrogen bonding, but they overlap with those of CMC; thus, the spectrum of sample B1 appears similar to that of sample A1.

Sample A3 is a 50:50 blend of CMC and CSM. The FT-IR spectrum contains the CMC peaks and also the peaks due to CSM. As noted earlier [21,41], the CSM has peaks at 3700–3000 cm^−1^ due to the O–H and N–H stretching in the protein. Smaller peaks at 2917 and 2863 cm^−1^ correspond to CH2 asymmetrical and symmetrical stretching vibrations, respectively. The bands at 1628 and 1535 cm^−1^ correspond to amide I band (mainly amide C=O stretching) and amide II band (N-H in-plane bending and C–N stretching). The weak amide III bands are observed near 1230 cm^−1^ and involve CN stretching and NH bending. The band at 1404 cm^−1^ corresponds to carboxylate vibrations in glutamic and aspartic acids [42]. The bands around 1040 cm^−1^ are related to the C–O stretching in hydroxy-amino acids. In general, the observed spectrum of sample A3 is consistent with the presence of CMC and CSM. As for the FT-IR spectra for samples B3 and C3 (blends of CMC, CSM and glycerol), because the peaks for glycerol overlap with those of CMC, the FT-IR spectra of samples B3 and C3 are similar to those of sample A3.

### 3.4. TGA Analysis

**Figure 2:**
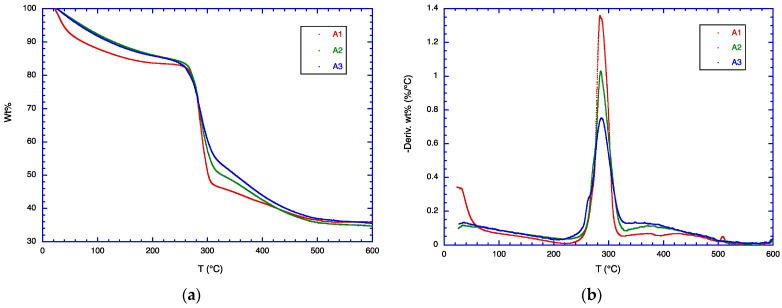
(**a**) TGA data and (**b**) DTG data for samples A1, A2, A3.

The TGA data for samples A1, A2, and A3 are shown in Figure 2. For sample A1 (pure CMC), the weight loss occurs in three stages. Thus, in the first stage, about 15% weight loss between 25 and 170 °C can be attributed to the loss of adsorbed water in CMC and possible dehydration reactions. In the second stage (around 220–350 °C), about 40% of the weight is lost due to degradation reactions, including the formation of smaller CMC fragments and some aromatic structures, and the loss of carbon dioxide and other volatile species. At 400 °C and above, further degradation occurs, leading to carbonaceous residues, more aromatic structures, and char, together with inorganic salts from the counterions present in CMC. These findings are consistent with earlier reports [43,44]. 

In contrast to CMC, CSM degrades between ca. 200 and 400 °C [21,45]. The addition of CSM to CMC (samples A2 and A3) broadens the second stage degradation of CMC. In the DTG curves, the curves for samples A2 and A3 are higher at 330–430 °C than for sample A1. Note that the weight loss during the first stage (<170 °C) for sample A1 is greater than that of samples A2 and A3, indicating that CMC contains more adsorbed water than CMC/CSM blends.

**Figure 3:**
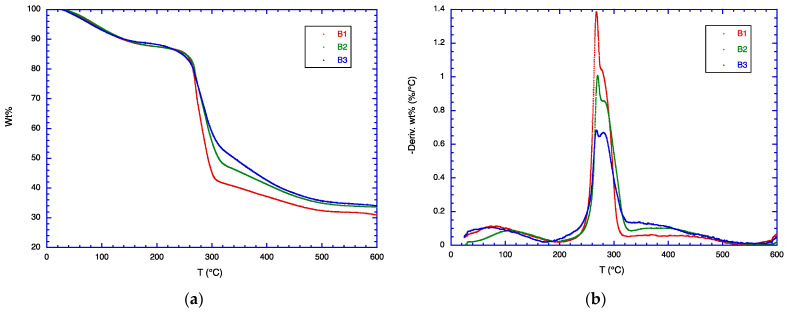
(**a**) TGA data and (**b**) DTG data for samples B1, B2, B3.

**Figure 4:**
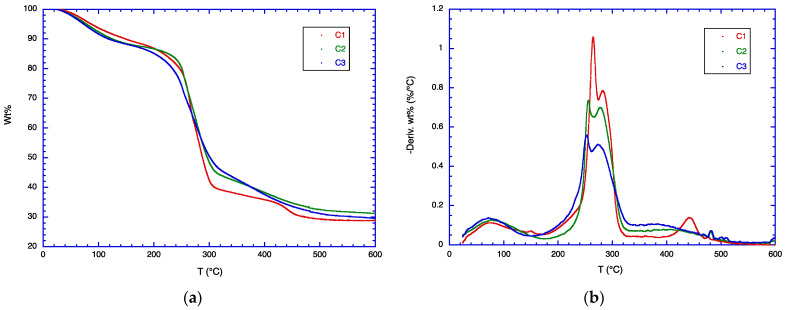
(**a**) TGA data and (**b**) DTG data for samples C1, C2, C3.

The TGA data for samples B1, B2, and B3 are shown in Figure 3 and for samples C1, C2, and C3 in Figure 4. These samples are similar to the samples A1, A2, and A3, except that 20 parts by weight of glycerol is added to each blend in the B series, and 40 parts by weight of glycerol in the C series. As in the A series, the effect of CSM addition in the B and the C series is to broaden the temperature range of the weight loss. This is observed clearly in the DTG curves, where the peak maximum decreases from pure CMC to CMC/CSM blends, and the DTG curve intensity is higher at 330–430 °C. Glycerol normally evaporates at ca. 200 °C, but in the blends it is hydrogen-bonded to both CMC and CSM, so that it does not exhibit a separate weight loss at 200 °C. As glycerol is added, the DTG curves are somewhat shifted to lower temperatures, become broadened, and also split into two peaks. This result suggests that glycerol exhibits different extents of plasticizer effects. All the blend molecules are impacted by glycerol, but the effect is not homogeneous, so that CSM may show different effects of glycerol plasticization from CMC.

### 3.5. Optical Microscopy

**Figure 5:**
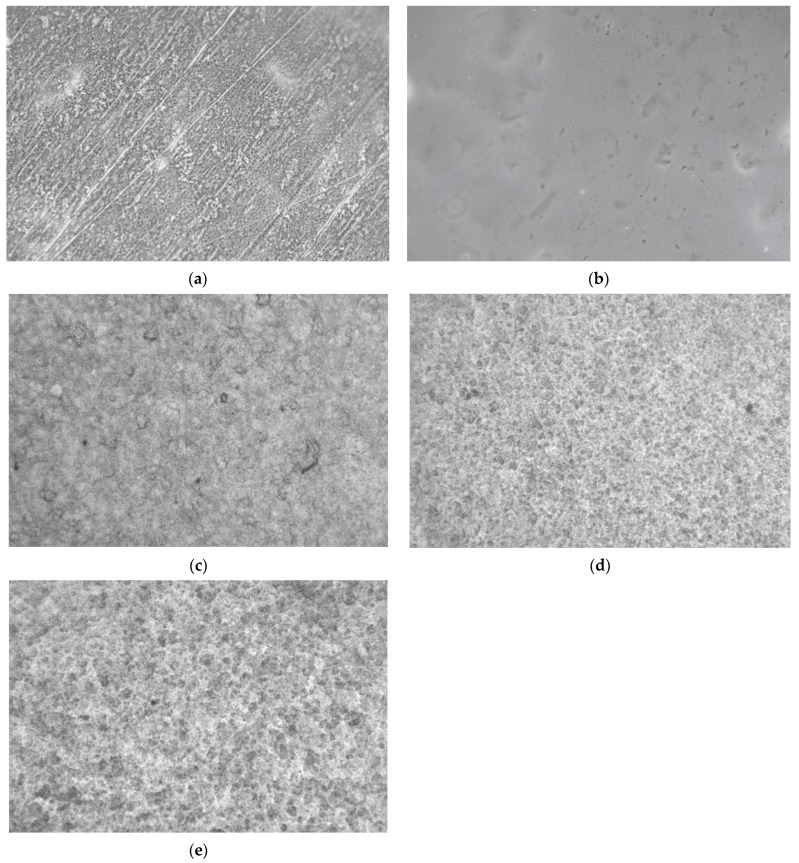
Optical photomicrographs of the surfaces of (**a**) sample A1; (**b**) sample B1; (**c**) sample A3; (**d**) sample B3; (**e**) sample C3. Magnification 44×.

Selected film samples were examined by optical microscopy. The optical photomicrograph of sample A1 is shown in Figure 5a. The image shows a slightly rough appearance due to the dried polymer. Sample B1 with CMC and glycerol (Figure 5b) shows an image with a smoother look due to the presence of glycerol. In contrast, the picture of sample A3 (CMC/CSM, Figure 5c) shows a more rugged morphology, consistent with a blend of two polymers. The corresponding image of sample B3 shows almost a network-like structure due to the interactions between CMC, CSM, and glycerol. In comparison, the image of sample C3 (with more glycerol) shows a rougher and more network-like appearance. 

### 3.6. Moisture Sorption Analysis

The propensity of the films to adsorb moisture was evaluated via a dynamic vapor sorption analyzer. By observing the weight loss of each sample after the 6 h hold at 0% RH, we can estimate the moisture content of four film samples at room temperature: A1, 9.12%; A3, 5.44%; B3, 3.78%; C3, 1.78%. It may be noted that the moisture content of sample A1 is larger than that of A3. Thus, the adsorption of moisture is better for CMC than for CSM. By comparing films A3, B3, and C3, it is observed that the moisture content of film decreases as the plasticizer content increases. This result suggests that the adsorption of moisture is driven primarily by the amount of CMC present, while the inclusion of plasticizer hampers this process. This observation is supported by the sorption isotherm analysis, as given below.

**Figure 6:**
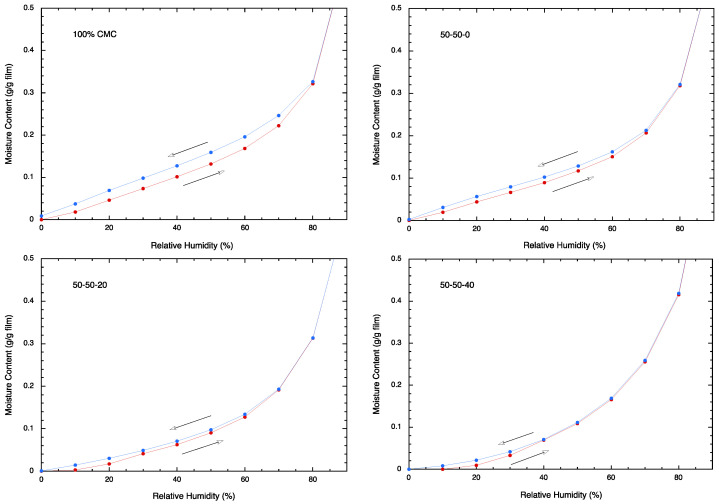
Moisture adsorption/desorption behavior of the CMC/CSM films, as shown by the moisture content (g moisture/g film) versus relative humidity. The arrow gives the direction of the increase or decrease of the humidity level.

The moisture adsorption/desorption behavior for the CMC/CSM/glycerol films is rather similar for all samples (Figure 6). Each of them shows a monotonous increase in adsorption of moisture during the adsorption process and the releases of the moisture during the desorption process. Sample A1 (100% CMC) picks up moisture quicker than the other films during the adsorption cycle and does not readily release moisture during the desorption process. This behavior can easily be noticed in the sorption isotherm. The retained moisture can be identified by the difference in the moisture content at each humidity value during the adsorption/desorption cycle. Since sample A3 (50:50 CMC/CSM) shows far less retained moisture, it is concluded that the surface adsorption of these films is mainly due to the presence of CMC. By comparing samples A3, B3, and C3, it is noticed that the retained moisture decreases as more plasticizer is added to the film. Therefore, these data confirm the earlier suggestion that the adsorption of moisture is positively affected by the CMC and negatively by the plasticizer (i.e., glycerol) in the film.

The moisture sorption data for CMC/CSM blends are similar to the results obtained for CMC/soy protein blends [34], where higher CMC contents led to higher moisture sorption. However, for CMC/gelatin blends [30], higher gelatin contents led to higher moisture absorption because gelatin is more hydrophilic than CMC. 

### 3.7. Swelling Test

**Figure 7:**
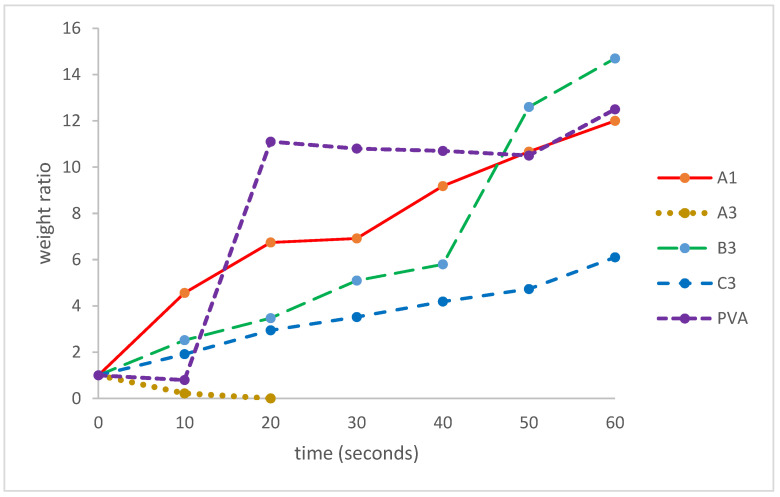
Water swelling data (weight ratios for swollen film versus dry non-swollen films).

Film samples A1, A3, B3, and C3 were also subjected to the water swelling test, together with a sample of poly(vinyl alcohol) (PVOH). The results are summarized in Figure 7. Sample A3 (50:50 CMC/CSM) dissolved and disintegrated within 20 s. The addition of glycerol enhanced the durability of the films in water. In fact, except for sample A3, all other samples swelled to several times their weights up to a soaking time of 60 s (after 60 s, some films felt like watery slimes, and the weights were difficult to determine). Among the samples swollen at 60 s, sample B3 (with 20% glycerol) showed the highest weight ratio of 14.7, followed by PVOH at 12.5, sample A1 at ca. 12, and sample C3 at 6.1. Thus, the four film samples A1, A3, B3, and C3 show variable behavior in water swelling; in particular, samples B3 and A1 exhibit somewhat similar swelling behavior as PVOH. 

### 3.8. Comments

PVOH is often used [25,46,47] for water-soluble food packaging and coatings and as dissolvable bags and pouches for detergents and agrochemicals. However, PVOH is obtained from the hydrolysis of poly(vinyl acetate), which is derived from petroleum. The trend today is to reduce the reliance on petroleum-based materials due to their environmental impact, finite resources, health concerns, and waste management [48]. Instead, agro-based water-soluble polymers (which are more sustainable, renewable, and environmentally friendly) are preferable [48,49]. As noted earlier, CMC and CSM are both agro-based and biodegradable. Since the CMC/CSM blends have similar water swelling properties as PVOH, they may be good candidates for use as replacement for PVOH. 

A concern for the use of bio-based materials is the price. Currently, PVOH sells for about USD 1.10–1.50/lb [50]. CMC is somewhat more expensive, at about USD 1.50–2.00/lb [51]. Cottonseed meal (dry basis) is priced at about USD 0.42/lb [52]. Thus, the blend of CMC/CSM should be cost-competitive relative to PVOH. 

In addition to their use in packaging and coatings, water-soluble polymers have been studied for a range of other applications, e.g., thickeners, defoamers, binders, electrolyte solvents, dispersants, stabilizers, surfactants, laxatives, and excipients [53], oil sand and heavy oil industrial use [54], and home care, water treatment, and crop protection [55]. Most of these applications currently involve synthetic water-soluble polymers. It is likely that agro-based materials (like CMC, cottonseed protein, and their blends) may be considered for these applications in the future.

## 4. Conclusions

In view of the environmental challenges posed by synthetic plastics in food packaging, we have been attracted to the use of agro-based and biodegradable materials, such as cellulosic derivatives and cottonseed protein. In this work, the feasibility of making films from the blends of CMC and CSM has been demonstrated. The addition of glycerol has been shown to improve the elongation at break. The addition of CSM to CMC reduces the mechanical properties of the films, but at a 75:25:40 ratio of CMC:CSM:glycerol, the elongation at break is maximized, whereas the tensile strength is about average. The addition of CSM to CMC does have the benefit of reducing the water vapor permeation, increasing the opacity of the films, relative to CMC alone, and decreasing the film cost. 

As both CMC and CSM are water-soluble, these blends may possibly be utilized as water-soluble food packaging and coatings and as dissolvable bags and pouches for food items or for laundry detergents, cleaners, and agrochemicals. In view of these potential applications, water swelling and moisture sorption tests were conducted on the CMC/CSM film samples. The results indicate that these films have different susceptibility to water; thus, depending on the application being considered, a particular blend composition may be chosen for consideration. 

There is increasing interest in the byproducts of cotton [13,56,57]. As CMC can be derived via cellulose from cotton linters and CSM from cottonseed, the use of these materials is beneficial to the cotton industry and potentially adds value to cotton production.

## Figures and Tables

**Table 1:** Dry weight ratios for the CMC/CSM blends.

Sample Code	CMC	CSM	Glycerol
A1	100	0	0
A2	75	25	0
A3	50	50	0
B1	100	0	20
B2	75	25	20
B3	50	50	20
C1	100	0	40
C2	75	25	40
C3	50	50	40
C4	25	75	40
C5	0	100	40

**Table 2:** Young’s modulus (YM), tensile strength (TS), and elongation at break (EB) of the CMC:CSM blend films.

Code	Weight Ratio CMC:CSM:Glycerol	Thickness (mm) *	YM (MPa) *	TS (MPa) *	EB (%) *
A1	100:0:0	0.0751 ± 0.0053 ^b^	3909 ± 178 ^a^	56 ± 4 ^a^	5 ± 0 ^d^
A2	75:25:0	0.0798 ± 0.0037 ^b^	3911 ± 105 ^a^	50 ± 6 ^a^	3 ± 1 ^d^
A3	50:50:0	0.0524 ± 0.0027 ^d^	3710 ± 143 ^a^	33 ± 3 ^b,c^	1 ± 0 ^d^
B1	100:0:20	0.0725 ± 0.0133 ^b,c^	2080 ± 361 ^b^	40 ± 6 ^b^	12 ± 2 ^c^
B2	75:25:20	0.0731 ± 0.0130 ^b,c^	1159 ± 116 ^c^	27 ± 1 ^c^	21 ± 4 ^b,c^
B3	50:50:20	0.0719 ± 0.0037 ^b,c^	1763 ± 102 ^b^	24 ± 1 ^c^	3 ± 0 ^d^
C1	100:0:40	0.0806 ± 0.0110 ^a,b^	660 ± 52 ^d^	23 ± 3 ^c^	34 ± 3 ^a,b^
C2	75:25:40	0.0812 ± 0.0114 ^a,b^	309 ± 29 ^e^	20 ± 1 ^c^	51 ± 2 ^a^
C3	50:50:40	0.0993 ± 0.0122 ^a^	164 ± 44 ^f^	12 ± 2 ^d^	50 ± 5 ^a^
C4	25:75:40	0.0720 ± 0.0042 ^b,c^	65 ± 7 ^g^	6 ± 1 ^e^	54 ± 9 ^a^
C5	0:100:40	0.0744 ± 0.0083 ^b^	51 ± 8 ^g^	2 ± 1 ^e^	51 ± 11 ^a^

* The data in each column were subjected to analysis of variance using the Tukey test method; according to this analysis, the same superscript letter for two or more values indicates that these values are not significantly different at *p* = 0.05.

**Table 3:** Water vapor permeation (WVP) and opacity of bilayer films of blends of CMC and CSM.

Code	Weight Ratio CMC:CSM:Glycerol	Thickness (mm) *	WVP (mg-m/kPa-d-m^2^) *	Thickness (mm) *	Opacity (A/mm) *
A1	100:0:0	0.0547 ± 0.0027 ^d^	0.894 ± 0.067 ^a^	0.0668 ± 0.0191 ^a,b,c,d^	1.2 ± 0.5 ^d^
A2	75:25:0	0.0683 ± 0.0070 ^a,b,c^	0.459 ± 0.048 ^b^	0.0877 ± 0.0056 ^a^	18.0 ± 1.3 ^c^
A3	50:50:0	0.0560 ± 0.0022 ^d^	0.310 ± 0.017 ^d,e,f^	0.0607 ± 0.0072 ^c,d^	28.0 ± 1.9 ^a^
B1	100:0:20	0.0620 ± 0.0055 ^b,c,d^	0.371 ± 0.016 ^c^	0.0703 ± 0.0281 ^a,b,c,d^	0.9 ± 0.2 ^d^
B2	75:25:20	0.0596 ± 0.0213 ^a.b.c.d^	0.320 ± 0.041 ^c,d,e,f^	0.0803 ± 0.0095 ^a,b^	17.6 ± 0.8 ^c^
B3	50:50:20	0.0683 ± 0.0047 ^a,b,c^	0.180 ± 0.014 ^g,h^	0.0841 ± 0.0130 ^a,b^	21.0 ± 3.5 ^b,c^
C1	100:0:40	0.0905 ± 0.0209 ^a^	0.325 ± 0.049 ^c,d,e,f^	0.0727 ± 0.0111 ^a,b,c^	1.0 ± 0.3 ^d^
C2	75:25:40	0.0721 ± 0.0106 ^a,b,c^	0.269 ± 0.037 ^d,e,f,g^	0.0809 ± 0.0260 ^a,b^	19.7 ± 5.0 ^b,c^
C3	50:50:40	0.0683 ± 0.0192 ^a,b,c,d^	0.227 ± 0.033 ^f,g,h^	0.0711 ± 0.0155 ^a,b,c,d^	23.5 ± 5.3 ^a,b^
C4	25:75:40	0.0767 ± 0.0160 ^a.b.c^	0.296 ± 0.019 ^d,e,f^	0.0692 ± 0.0069 ^b,c,d^	21.5 ± 1.9 ^b^
C5	0:100:40	0.0750 ± 0.0326 ^a,b,c,d^	0.319 ± 0.048 ^c,d,e,f^	0.0593 ± 0.0014 ^c,d^	23.3 ± 6.2 ^a,b^

* The data in each column were subjected to analysis of variance using the Tukey test method; according to this analysis, the same superscript letter for two or more values indicates that these values are not significantly different at *p* = 0.05.

## Data Availability

Data are contained within the article.
